# The “Elderly” Lesson in a “Stressful” Life: Italian Holistic Approach to Increase COVID-19 Prevention and Awareness

**DOI:** 10.3389/fendo.2020.579401

**Published:** 2020-09-30

**Authors:** Sabrina Angelini, Alessandro Pinto, Patrizia Hrelia, Marco Malaguti, Fabio Buccolini, Lorenzo Maria Donini, Silvana Hrelia

**Affiliations:** ^1^Department of Pharmacy and Biotechnology, University of Bologna, Bologna, Italy; ^2^Experimental Medicine Department, Sapienza University of Rome, Rome, Italy; ^3^Department for Life Quality Studies, University of Bologna, Rimini, Italy; ^4^3C Engineering Srls, Rome, Italy

**Keywords:** COVID-19, PM10, nutrition, oxidative stres, resilience

## Abstract

It's a frightening time due to COVID-19, but the great elderly/centenarians, apparently with more frailty, seem to have a better response to the pandemic. “The South Italy” lifestyle seems an “effective strategy” promoting the well-being embedded in a holistic solution: healthy diet, less exposure to PM10 pollution, protected environment, and moderate physical activity. The European FP7 Project RISTOMED results, since 2010, have shown that dietary intervention improved a heathy status in the elderly people. Based on the RISTOMED results, in addition to sociocultural and environmental factors, the authors suggest an integrated approach for resilience to COVID-19. Such an approach during the next months could make the difference for the success of any government progress policy to fight COVID-19, finalizing long-term well-being and successful aging.

## Introduction

Coronavirus disease 2019 (COVID-19) is a highly infective, respiratory disease caused by SARS-CoV-2, identified for the first time in Wuhan, China. At the end of February 2020, the first case was reported in Lombardy (Italy), opening an unforeseen sanitary crisis in Europe and later on all over the world. On June 29, 2020, there were ~240,400 confirmed cases of COVID-19 and almost 34,700 deaths recorded in Italy^1^. About 10% of COVID patients develop an acute respiratory distress syndrome (ARDS), which represents the leading cause of death among these patients (1). COVID-induced ARDS leads to lung histopathologic changes, including diffuse alveolar damage, chronic inflammatory infiltrates, and intra-alveolar fibrinous exudates (2). Like in any viral infections, the immune system triggers the production of reactive oxygen species (ROS) and reactive nitrogen species (RNS) that are directly involved in the development of lung fibrosis and decrease of lung function (3, 4). Recently, one possible origin of the diffusion in our country has been in the Italy-China trade (5). In particular, Bontempi identified a gradient from north to south Italy, with Lombardy, the Italian region most affected by COVID-19, as the most involved in commercial relationship with China. However, the first confirmed cases reported in Italy originated from Munich, (Germany). Nevertheless, this seems to support the importance of the commercial factor. Indeed, in 2019—when probably the SARS- CoV-2 was already circulating—Germany has been the first European destination country for Chinese goods, while Italy represented the 21st market for Chinese export^2^. Therefore, we can assume that the economic relationship with China could have had a role in the initial diffusion, whereas other features intervened in the rapid spread of the disease. Available data describe that symptom severity and mortality rates are higher among elderly COVID-19 patients than in younger patients (6). According to the report of the Italian National Institute of Statistics^3^, breaking down the excess mortality by age groups, it has been observed that COVID-19's contribution to mortality decreases as the age increases, passing from 78.5% of the excess in the 50–59 years old class to 24% in >90 years old. Moreover, according to the report on infections diagnosed by Italian regional reference laboratories on May 4 (at the end of the Italian lockdown)^4^, we observed a north to south decreasing gradient in the percentage of > 90 being infected (from 6 to 8% in the northern regions and from 1 to 4% in the southern regions). Thus, it seems that very old people and centenarians might be more resilient to COVID-19 infection, particularly if they live in South Italy regions. To explain this paradox, we hypothesized the influence of air pollution, nutrition, overweight/obesity, and aging as the possible factors for a successful aging and counteracting an “*unknown agent*” as SARS-CoV-2. In particular, we focused on oxidative stress, metaflammation, and the capability of immune system to cope with a variety of stressors, assuming that a better efficiency and balanced activity of the immune system, therefore a better resilience to stress, are subordinate to a healthy environment, nutrition, and lifestyle beyond the genetics. Furthermore, we also refer to the results of the European FP7 Project RISTOMED conducted on elderly subjects by our research group, which seem to support our hypothesis.

## Air Pollution and Covid-19: The Italian Experience

Efforts to identify effective therapeutic drugs and treatments has been hampered by our limited understanding of the host's immune response to the disease (7). In particular, the observed aberrant inflammatory response—the so-called “cytokine storm” —occurring during COVID-19 infection is still not clear. In this uncertainty, chronic exposure to particulate matter (PM), ozone, and other pollutants is known to affect respiratory function, and recently, environmental pollution has been associated to ARDS (8–11), which is one of the features of SARS-CoV-2 infection. Furthermore, environmental pollution, mostly PM, is clearly emerging as an important factor and effective vehicle of infection. In 2015, the World Health Organization (WHO) clearly defined, for the first time, environmental pollution, particularly PM10 and PM2.5, as the world's largest health risk factor (12). Not surprisingly, therefore, the 2020 COVID-19 pandemic was extremely severe in regions around the Po Valley–Lombardy, Emilia-Romagna, and Veneto, recognized as one of the most polluted geographical areas in Europe (13). Indeed, according to the “2019 air quality analysis,” 54 cities have exceeded the limit set for PM10 and/or ozone; interestingly, the top 25 positions are all occupied by cities in the Po Valley, and all have passed both quality indicators, while southern cities are involved only in exceeding the limits for ozone^5^. The underlying hypothesis is that a high concentration of PM makes the respiratory system more susceptible to COVID-19 and related complication. According to studies on previous viral infections (i.e., H5N1, RSV, and measles), PM represents an effective carrier for viruses transport and diffusion and viral disease proliferation as well (14, 15). This also applies to COVID-19, and a recent position paper by the Italian Society of Environmental Medicine has proposed a possible link between the high mortality rates observed in Northern Italy due to COVID-19 and the PM concentrations (16, 17). Additionally, a significant association has been found between geographical distribution of daily exceeding of PM10 limits (law limit 50 μ/m^3^) in 110 Italian provinces and COVID-19 spreading, before the lockdown decided by the government (15). Although suffering from some methodological limitations, this pioneering study highlighted a more frequent number in PM10 exceedances in Lombardy and in cities located in the Po Valley than those observed in Rome and Southern Italy, where the diffusion and lethality of the virus was significantly lower compared to those of northern regions (16, 17). This hypothesis is further supported by recent studies reporting that long-term exposure to environmental pollution may enhance susceptibility to severe SARS-CoV-2 infection in the United States (18, 19).

**Figure 1:**
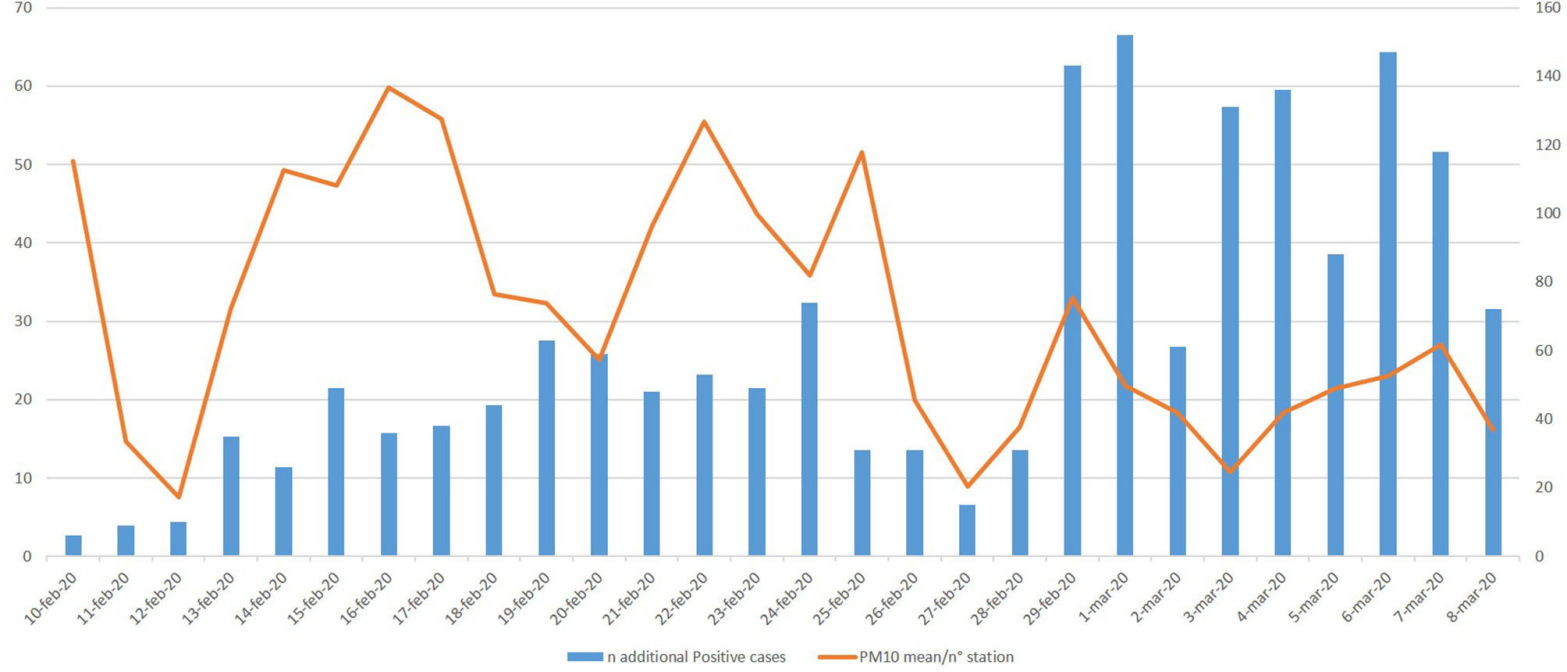
Piacenza as a case study—mean PM10/n° station from February 10 to March 8 (data from: https://apps.arpae.it/qualita-aria/bollettino-qa-provinciale/pc) and new COVID-19 positives 14 days later the environmental PM10 detection. * The high number of infected subjects registered from February 29 is attributable to the greater number of swabs made. However, the trend of the infected peaks follows that of PM10.

In this context, we considered the PM10 daily concentration and COVID-19-infected subjects in the city of Piacenza, taken as a case study (Figure 1). We plotted together the mean level of PM10 (collected by ARPA, and expressed as mean μ/m^3^/number of station) from February 10 up to the day of the Italian lockdown, and COVID-19-infected subjects identified from February 24 up to March 22 (14 days later the PM10 detection: a delay that takes into account the maximum incubation period). The observed trend leads us to hypothesize a relationship between PM10 and COVID-19 infection. Overall, these early studies on COVID-19 suggest that pollution, PM in particular, could act directly as a carrier and indirectly as an amplifier of the effects on the lung and bronchi of the COVID-19. Therefore, monitoring PM10 concentration should become even more important in view of the unpredictable future related to COVID-19.

## Mediterranean Diet and Covid-19: The Italian Experience

The interaction between antigens, from both endogenous and exogenous sources, and the immune system results in the release of ROS and RNS, leading to the activation of transduction signaling pathways that induce the expression of pro-inflammatory cytokines and chemokines, that consequently, promote further ROS and RNS generation, leading to a vicious circle (20). It has been proposed that inflammaging, a condition defined as the combination of a chronic inflammatory state together with aging, is involved in numerous diseases that commonly affect elderly population. Inflammaging is associated with an increased level of ROS, which can be responsible for oxidative stress, damages at different cellular macromolecules and organelles, further increased inflammation, and activation of cell death pathways (21). It appears evident that oxidative stress represents a key factor in the development of chronic inflammation. However, data from studies on >90 years old and centenarian people demonstrated that most of these people have escaped or delayed chronic and degenerative diseases despite high plasma levels of inflammatory cytokines, acute phase proteins, and coagulation factors (22). This paradox has been explained considering that centenarians, besides being strongly inflamed, also present high transforming growth factor (TGF)-b1, adiponectin, cortisol and other molecules characterized by marked anti-inflammatory action. Ultimately, it appears that a balance between proinflammatory and anti-inflammatory factors matters more than simply to have a low inflammatory status, for a long and healthy life (23).

The EU FP7-SME Project RISTOMED (new E-services for a dietary approach to the elderly) evaluated the impact of a personalized diet on inflammatory biomarkers, nutritional status, oxidative stress, and intestinal microbiota in healthy elderly people. The RISTOMED project aimed to use diet to improve health-related quality of life for elderly and to prevent age-related diseases. The project promoted an optimized diet able to deliver the daily requirement of each nutrients, defined by WHO, to elderly people, and was designed to reduce inflammation and oxidative stress (24). In particular, results on oxidative stress biomarkers evidenced in elderly people a positive effect of a healthy diet, which was able to increase plasma antioxidant and detoxifying enzyme activity such as superoxide dismutase (SOD) and glutathione S-transferase (GST).

What can we learn from these results and how can we apply them to the worse outcomes observed in COVID-19 pandemia?

As previously stated, COVID-19 infection can generate a mild or highly acute respiratory syndrome with a consequent release of pro-inflammatory cytokines, including IL-6, TNFα and, consequently, an increase in the intrapulmonary oxidative burden (25). In many diseases, the redox balance, or rather the balance between oxidants and antioxidants, is altered with severe consequences. ROS/RNS imbalance and overproduction are involved in the development of various type of stresses such as oxidative, nitrative, carbonyl, inflammatory, and endoplasmic reticulum stress, which, in turn, lead to inflammation and an altered immune response at lung level. In this context, antioxidants appear as important agents in the counteraction of lung oxidative stress (26).

Recently, Abouhashem et al. demonstrated that the SOD3 gene is downregulated in the elderly and proposed a new hypothesis to understand why elderly are more likely to suffer lung complications in COVID-19 (31). Following previous studies, evidencing that SOD administration can decrease the severity of respiratory illness, the authors propose to better evaluate if lung-specific delivery of SOD3 related antioxidants, in combination to other anti-virals, may improve COVID-19 outcomes in the elderly. Moreover, Horowitz et al. reported that both oral and intravenous glutathione, N-acetyl-cysteine (a well-known glutathione precursors) may represent a novel approach for addressing respiratory distress and “cytokine storm syndrome” by blocking nuclear factor-kappaB (NF-κB) in patients with COVID-19 pneumonia (32). Glutathione is a well-known endogenous antioxidant well-distributed in most tissues where it acts as both an intra- and extracellular antioxidant. In particular, glutathione is abundant in the airway epithelial lining fluid, where it provides the first line of defense against oxidative stress, e.g., caused by air pollution, and helps to decrease inflammatory lung processes. Both SOD and GST synthesis are modulated by the nuclear factor erythroid 2-related factor 2 (Nrf2), a fundamental regulator of antioxidant and detoxifying enzymes. Nrf2, as a transcription factor, modulates the expression of numerous antioxidant-response-element-dependent genes to regulate the physiological and pathophysiological outcomes of oxidant exposure (33). Polyphenols, in particular flavonoids and isothiocyantes, such as sulforaphane, are well-known Nrf2 activators and are abundantly present in the Mediterranean diet (34). Adherence to the Mediterranean diet in Italian population was higher in the south than in the north, and in elderly populations compared to younger people in which a slow but constant abandonment of traditional dietary and lifestyle patterns has been observed (35). Conversely, the Western dietary pattern [high-energy density with low micronutrient content; high fat intake, particularly saturated long-chain fatty acids and trans fatty acids; high assumption of salt (sodium chloride), “free sugars,” and foods providing “empty calories” (e.g., sugar-sweetened drinks); poor in dietary fiber and phytochemicals due to low intake of whole cereals, fruits, and vegetables, and conversely rich in refined cereals; higher glycemic index and glycemic load] is able to amplify the chronic low-grade systemic inflammation. A long-term high-fat diet induces oxidative stress both in animal models and in humans: it is hypothesized that mitochondrial dysfunction, induced by NADPH oxidase activity and by increased fatty acid oxidation, may contribute to oxidative stress. ROS and lipid peroxidation deplete vitamins and antioxidant enzymes hampering the oxidative stress (36). Strong evidences suggest that long-chain saturated fatty acids can boost proinflammatory signaling by binding to the toll-like receptors, TLR4 and TLR2, which in turn activate the NF-κB signaling cascade (37). Advanced glycation end-products also induce oxidative stress, activating the ubiquitous plasma membrane redox system (PMRS), which transfers electrons from intracellular donors (NADPH, ascorbate) to extracellular acceptors. This PMRS action is linked to the generation of superoxide radicals and other ROS/RNS by NADPH oxidase (38). Furthermore, the excessive energy intake, in particular as fatty acids and glucose, leads to a chronic metabolic stress involving the endoplasmic reticulum stress (ERS), directly or through the mitochondrial ROS overproduction. The ERS activate the unfolded protein response to maintain the ER homeostasis but the unfolded protein response cascade, via NF-κB pathway, leads to the secretion of TNF-α, IL-6, and IL-1β, by metabolic cells such as adipocytes, myocytes, and hepatocytes (36).

Furthermore, obesity, in particular abdominal obesity, associated with an excessive energy intake and to a Western-type dietary pattern, join together to produce a chronic low-grade systemic inflammation. Hotamisligil coined the term metaflammation to design this status of metabolic inflammation (27). In overweight/obesity, the increased adipocytes size is associated with tissue hypoxia, leading to the production of pro-inflammatory adipokines and cytokines (such as TNF-α, IL-1, and IL-6) and to the shift of macrophages resident in adipose tissue (AT) from anti-inflammatory polarized M2 to pro-inflammatory M1 phenotype (39). Through the appearance of the adipokines in bloodstream, the AT inflammation is “transferred” to other tissues, such as liver, pancreas, hypothalamus, and skeletal muscle, leading to onset of insulin resistance and metabolic syndrome onset and impaired immune response in the lung parenchyma and bronchi (27, 40). Several epidemiological evidences along with a consistent pathogenetic rationale lead to speculate that obesity *per se* may be an independent risk factor for SARS-CoV-2 (27). The main hypothesized mechanisms linking obesity to susceptibility to SARS-CoV-2 infection are summarized in Table 1.

**Table 1:** Main hypothesized mechanisms linking obesity to susceptibility to SARS-CoV-2 infection.

Obesity is recognized as an independent risk factor for more severe and longer duration infection with a worse prognosis; this condition was observed during the influenza A H1N1 epidemic.	(27, 28)
Obesity, particularly abdominal obesity, resulting in a restrictive lung syndrome, is an independent risk factor for hypoventilation syndrome in Intensive Care Unit patients, leading to oxygen blood desaturation and to respiratory failure in acute respiratory distress syndrome (ARDS).	(27, 28)
The presence of fat droplets within the alveolar interstitial space in obese diabetic rats and ectopic adipocytes within the lung parenchyma observed in a small population sample correlate with the inflammatory infiltrate contributing to the massive interstitial edema in ARDS.	(29)
Viruses express a specific tropism for different tissues and cell types including adipocytes (H1N1, Type A influenza, and adenovirus-36), adipo-stromal cells (Adenovirus-36, CMV), endothelial cells (SARS-CoV-2), macrophages (influenza A, SARS-CoV, adenovirus-36, HIV), and lymphocytes (SARS-CoV-2, HIV). Currently, there are no clear evidences for direct SARS-CoV-2 infection of AT. If this hypothesis will be confirmed, the virus may induce an extensive activation of the signaling pathways for AT cytokines production.	(28, 29)
It has been recently proposed that the COVID-19 uses an angiotensin-converting enzyme 2 (ACE2)-dependent mechanism of cellular entry, similar to SARS-CoV and human respiratory coronavirus NL63. The interaction between the ACE2-RAS system, AT, and COVID-19 could explain the higher morbidity and mortality risk for COVID-19 patients with obesity. The role of ACE2-RAS in COVID-19 remains to be elucidated.	(29, 30)
Human dipeptidyl peptidase 4 (DPP4) expression is higher in visceral AT and directly correlates with adipocyte inflammation and insulin resistance. DPP4 plays also an important role in immune regulation by activating T cells and upregulating CD86 expression and NF-jB pathway. DPP4 was also identified as a functional receptor for the spike protein of the Middle East respiratory syndrome (MERS)-CoV. DPP4 could also play a role in the immune response to COVID-19.	(30)
Excess fat is associated with a complement system over activation. The complement system was identified as an important host mediator of SARS-CoV-induced disease.	(29)

The “pre-activation” of inflammatory response in the AT of overweight or obese subjects makes this organ a potential target for further immune response amplification by exogenous pathogens such as viruses (28, 29, 41, 42). The imbalance between anti- and pro-inflammatory adipokines secretion from thoracic visceral fat depots, such as the epicardial and mediastinal, can play a role in the cytokine storm described in patients with severe SARS-CoV-2 (30). In early COVID-19 studies, IL-6 was a strong independent predictor of mortality, and adiponectin was reported to predict mortality in critically ill patients upon admission to the Intensive Care Unit (ICU) (28, 29). Moreover, in the elderly it is worth noting that aging causes visceral fat accumulation, AT inflammation, and fibrosis (30). In conclusion, the better adherence to the Mediterranean diet of very old people in South Italy could explain their resilience to COVID-19 and suggests that a dietary regimen modification in order to improve the nutritional assumption of Nrf2 activators might be useful both to prevent pulmonary complications and to improve their outcomes (26).

## Conclusion

In May 2020, Italy was at the end of the COVID-19 pandemic emergency, and we will have to live with the virus with relevant health, socioeconomic, and political consequences. Therefore, to avoid the spread of a novel emergency and lethal infection in the near future, we should urgently adopt actions to counteract environmental pollution and promote healthy nutrition because we are still in the absence of effective therapeutic tools/vaccine. Indeed, as discussed, people aged ≥90 years might be more resilient to COVID-19 infection, and their “secret” could be highly related with their lifestyles over the past years. In particular, living in less polluted environments and respecting long life healthy eating habits with a high level of adherence to the Mediterranean diet could represent the winning weapon. Overall, all the early studies on COVID-19 suggest that pollution, PM in particular, could act directly as a carrier and indirectly as an amplifier of the effects on the lung and bronchi of the COVID-19. Therefore, monitoring PM concentration should become even more important in view of an unpredictable future related not only to COVID-19. Nationwide lockdown measures adopted to counteract COVID-19, with a reduction in economic activities and road traffic, have highly contributed to lower environmental pollutant emission and possibly to defeat COVID-19 infection. Air pollution has proven to be an important factor that affects COVID-19 transmission and mortality rate in several countries (43–46). Northern Italy, where the air is more polluted than the rest of the country, was hit relatively hard by COVID-19, with significantly higher incidence and related death rate. This is a worldwide debated topic, and the National Institutes of Health and the Italian Institute for Environmental Protection and Research with the National System for Protection Environment have launched an epidemiological study at the national level to assess whether and to what extent air pollution is associated with the health effects of the epidemic. If this is confirmed, green policies, with concrete changes in different sectors—including energy, agriculture, food, housing, and transportation—should be promoted in order to preserve biodiversity and safeguard mankind accordingly with the international agreement and institutions such as IPCC, FAO, EU Commission, and US EPA.

At the same time, the eating habits in elderly (65–85 years), as deeply studied within the FP7-RISTOMED Project since 2008, showed that the Mediterranean Dietary Model positively influenced, even in the short and medium term, the oxidative and inflammatory phenotype. The anti-inflammatory effects of the Mediterranean Dietary Model could be mediated by epigenetic mechanisms already operating from *in-utero* life and early years of postnatal life, although a further analysis is needed to corroborate this hypothesis (34). Epigenetic factors are heritable from cell to daughter cell within the same organism, and there is growing evidence that this heritability can be transgenerational among organisms (47). Moreover, a recent study concluded that a 1-year Mediterranean-like-nutritional intervention can promote epigenetic rejuvenation in the elderly (48). Overall, adherence to the Mediterranean diet of people aged ≥90 years in South Italy could explain their resilience to COVID-19. In addition, it points out that a dietary regimen modification aimed at improving the nutritional assumption of Nrf2 activators might be useful both to prevent multi-organ complications and to take care of patients, improving their outcomes (26).

In our holistic vision, the binomial preventive combination of a non-polluted environment and Mediterranean Dietary Model should be available to all people to take advantage of its benefits, increasing their resilience. Indeed, a healthy lifestyle may have a great impact on a person's immune system, counteracting the rapid turn from an allostasis state (consisting in an imbalance of primary mediators due to inadequate environmental factors) to an allostatic load in the presence of an acute event, such as a viral infection. Therefore, in our opinion, a less-polluted environment and the Mediterranean Dietary Model represent a strategic preventive tool to successfully cope with COVID-19, as we learned from the “Italian elderly lesson.” Changing the approach according to the elderly lesson to be handed down to the future generation can make the difference for the success of any government progress policy not only to fight COVID-19 but also to finalize well-being and successful aging. Overall, this study has some limitation as, with regard to pollution, we limited our consideration to PM10, without considering other pollutants; furthermore, we did not consider other meteorological factors, including temperature, humidity, rainfall, wind speed, and UV exposure. These factors have been investigated in a number of articles, with mixed results, requiring additional research to provide conclusive evidence [for a review see (44)]. The majority of the studies, including ours, emphasizes the impact of climate indicators, without considering other features like human mobility, population density, and grade of urbanization, as well as living conditions and domestic hygiene (49). Indeed, the latter might contribute to the poor air quality, while population density, in particular if associated with low cultural level, could make social distancing difficult. Unfortunately, there aren't enough data to confirm the hypothesis of the work. Many efforts are necessary to ascertain by interdisciplinary analysis the relationship between biomedical markers of disease (and morbility and mortality indices) and demo-environmetal factors as air pollution and population density. These correlations would throw more light on both the causes of the pandemia and the possibility of putting forward hypotheses of corrective actions suitable to slow down the course of the epidemic while waiting for a safe vaccine for the population.

Finally, reminding us of the Professor Henry Nix words “data does not equal information; information does not equal knowledge; and, most importantly of all, knowledge does not equal wisdom (50),” we agree that today, in our socioeconomic model, we have a huge amount of data, great number of information, but still a small puddle of knowledge and an odd “*droplet*” of wisdom.

## Data Availability Statement

The original contributions presented in the study are included in the article/supplementary material, further inquiries can be directed to the corresponding author/s.

## Author Contributions

All authors listed have made a substantial, direct and intellectual contribution to the work, and approved it for publication.

## Conflict of Interest

FB was consultant by the company 3Cengineering Srls. The remaining authors declare that the research was conducted in the absence of any commercial or financial relationships that could be construed as a potential conflict of interest.

## Footnotes

^1^Available online at: http://www.salute.gov.it/portale/nuovocoronavirus/dettaglioContenutiNuovoCoronavirus.jsp?area=nuovoCoronavirus&id=5351&lingua=italiano&menu=vuoto

^2^Available online at: https://www.istat.it/it/commercio-estero?comunicati.

^3^Available online at: https://www.istat.it/it/files/2020/05/Rapporto_Istat_ISS.pdf (accessed May 27, 2020).

^4^Available online at: https://www.epicentro.iss.it/coronavirus/bollettino/Bolletino-sorveglianza-integrata-COVID-19_20-maggio-2020_appendix.pdf (accessed May 22, 2020).

^5^Available online at: https://www.legambiente.it/wp-content/uploads/2020/01/Malaria-di-citta-2020.pdf
